# The effect of intrinsic physiological traits on diapause survival and their underlying mechanisms in an annual bee species *Bombus impatiens*

**DOI:** 10.1093/conphys/coaa103

**Published:** 2020-12-04

**Authors:** Erin Treanore, Etya Amsalem

**Affiliations:** Department of Entomology, Center for Chemical Ecology, Center for Pollinator Research, Huck Institutes of the Life Sciences, Pennsylvania State University, University Park, PA 16802, USA

**Keywords:** Diapause, nutritional ecology, bees, floral resources

## Abstract

In the face of insect declines, identifying phases of the life cycle when insects are particularly vulnerable to mortality is critical to conservation efforts. For numerous annual insect groups, diapause is both a key adaptation that allows survival of inhospitable conditions and a physiologically demanding life stage that can result in high rates of mortality. As bees continue to garner attention as a group experiencing high rates of decline, improving our understanding of how annual bees prepare for diapause and identifying factors that reduce survival is imperative. Here, we studied factors affecting diapause survival length and their underlying mechanisms using an economically and ecologically important annual bee species, *Bombus impatiens*. We examined how age and mass upon diapause onset correlate with diapause survival length, and the mechanistic role of nutrient acquisition and oxidative stress post pupal eclosion in mediating these effects. Our findings show that both age and mass were strong predictors of diapause survival length. Heavier queens or queens in the age range of ~6–17 days survived longer in diapause. Mass gain was attributed to increases in lipid, protein and glycerol amounts following pupal eclosion, and the ability to deal with oxidative stress was significantly compromised in older pre-diapause queens. Our results demonstrate that age-related shifts in bee physiology and timing of nutrient acquisition may both be critical factors driving diapause survival.

## Teaser

Insects can use diapause to cope with extremes in seasonal environments. However, diapause is a physiologically demanding life stage that can result in mortality. Here we used an annual social bee, *Bombus impatiens,* to examine how mass, age, nutrient acquisition and oxidative stress predict winter diapause survival and propose recommendations for conservation strategies.

## Introduction

Native bees provide essential pollination services to both agricultural and wild ecosystems (Losey *et al.*, 2006). In temperate regions, the majority are annual bee species, their life cycle characterized by a phase of diapause followed by mating, nest foundation, offspring production and dispersal (Free, 1993, Michener, 2007). The diapause phase allows annual bee species to survive unfavourable environmental conditions, such as periods of extreme temperature or a lack of floral resources, and to synchronize their phenology with conspecific individuals. However, diapause can be physiologically and metabolically demanding, resulting in reduced survival and adult fecundity (Hahn *et al.*, 2011). Numerous annual bee species continue to decline across their geographical ranges (Hamblin *et al.*, 2018, Kerr *et al.*, 2015, Koh *et al.*, 2016); thus, identifying the role of stressors during bottleneck phases in the life cycle is necessary for developing effective conservation practices.

Annual bees prepare for diapause primarily during the pre-pupal or adult stage (Danforth *et al.*, 2019) by exhibiting a suite of behavioural and physiological changes such as increased sequestration of nutritional reserves and resistance to environmental stress (Denlinger, 2002, Hahn *et al*., 2011). Both adult and larval bees obtain nutrition from floral resources: pollen that is the main source of proteins, lipids and micronutrients, and nectar, the main source of carbohydrates (Heinrich, 1979, T'ai *et al.*, 2000). These nutrients are primarily stored as lipid and glycogen reserves in the fat body tissue, which is the main organ for nutrient storage, breakdown and mediation of metabolic processes (Arrese *et al.*, 2010, Hahn *et al.*, 2007). Adults foraging for nutrition prior to diapause may face a trade-off between nutrient accumulation and time spent in the field until diapause onset. Delaying entrance to diapause may result in acquiring more nutrients but may also increase the metabolic costs associated with a late entrance to diapause. A longer foraging period requires energy, time, possible exposure to disease or predators and coping with stressors related to aging (Ellington *et al.*, 1990, König *et al.*, 1995, Taylor, 1980). Conversely, a quick entrance to diapause may result in insufficient accumulation of fat body reserves and further metabolic costs associated with longer diapause and diapausing in warmer fall temperatures (Bosch *et al.*, 2010, Taylor, 1980).

Survival of diapause is often linked with higher body mass and thus increased nutritional stores (Beekman *et al.*, 1998, Bogo *et al.*, 2017, Roberts *et al.*, 2004). Nutrients are likely acquired in the days following eclosion (Woodard *et al.*, 2019), but detailed empirical data on this time period is limited to a few studies in a limited number of species (Alford, 1969a, Roseler *et al.*, 1986). Of these nutrients, lipids are a critical fuel source during diapause. For example, bumble bee queens may deplete 80% of their fat body lipids during diapause (Alford, 1969b). However, other less explored nutrients may be utilized during diapause, particularly when environmental conditions change during the diapause period (Sinclair *et al.*, 2018). For example, leafcutter bee larvae (*Megachile rotundata*) first utilize lipids during diapause and later shift to utilizing amino acids and glycogen (Yocum *et al.*, 2005). Glycogen can also be broken down into the precursors for glycerol, an important cryoprotectant, during cooler periods of diapause (Joanisse *et al.*, 1995, Storey *et al.*, 1991). In addition to these nutrients, increases in proteins, such as heat shock and storage proteins, are often associated with diapause preparation (Denlinger, 2002). Hexamerins (amino acid storage proteins and their associated transcripts) are often accumulated in the hemolymph prior to diapause in numerous insect species (Denlinger, 2002), including bumble bee queens (Colgan *et al.*, 2011). Energetic dynamics of diapause are complex, and multiple nutrients are likely necessary for diapause maintenance.

Annual bees preparing to diapause must balance nutrient acquisition with environmental threats and shifts in their own physiological quality (e.g. age). Therefore, the timing of two key events can have significant consequences for an individual’s diapause survival, these being (i) pre-diapause nutrient acquisition and (ii) timing of diapause onset. Acquiring sufficient nutrients depends on the local abundance and quality of floral resources; therefore, the synchronization of resource availability with the pre-diapause feeding period is critical. As habitat destruction and loss of floral resources are increasingly common and have been frequently implicated in bee declines, nutrients may be unavailable at this precarious timepoint (Goulson *et al.*, 2015). Timing of diapause onset can also significantly affect diapause survival. Older individuals that require a longer foraging period may experience declines in their physiological condition as they age. Aging was shown to affect the ability to store and use fat reserves, tolerate cold and respond to stress (Alford, 1969a, Bowler *et al.*, 2008, Lockett *et al.*, 2016). Furthermore, it has been shown in some bee species that the amount and composition of the fat body reserves change with age (Alford, 1969a, Votavova *et al.*, 2015) and older individuals have a limited thermal tolerance (Jensen *et al.*, 2007). In some social Hymenoptera females, older queens have shown reduced expression of antioxidant genes, which are critical for neutralization and protection against reactive oxygen species (ROS) (Corona *et al.*, 2005). Whether the antioxidant systems that protect against oxidative stress also shift their function with age is unknown. Altogether, the availability of floral resources, acquisition of nutrients, age of adult bees at the onset of diapause and the interactions between these factors may impact diapause survival.

Annual bees that undergo diapause as adults, such as some species in Augochlorini, Halictini and Bombini (Danforth *et al*., 2019, Santos *et al.*, 2019), are excellent systems in which to study the trade-off between aging and nutrient acquisition on diapause survival. In this study, we used the common eastern bumble bee *Bombus impatiens* to examine how queen age and mass affect queen diapause survival length and to quantify nutrient acquisition and oxidative stress as function of age prior to diapause onset. A previous study in *B. terrestris* demonstrated that queens below a specific mass threshold were unlikely to survive diapause, but higher mass did not improve diapause survival (Beekman *et al*., 1998). However, these queens varied in their age, which may explain the inconclusive results. Similar findings were demonstrated in previous studies (Bogo *et al*., 2017, Holm, 1972, Owen, 1988), all showing that heavier queens survived longer diapause in several bumble bee species; however, these studies all used weight categories and/or queens of an unknown age. Bumble bee distribution across higher latitudes and altitudes makes them crucial pollinators in alpine ecosystems and for numerous rare plants species, and their ability to buzz-pollinate makes them effective pollinators in many agricultural systems (Morandin *et al.*, 2001, Pleasants, 1980, Velthuis *et al.*, 2006). Furthermore, because bumble bee colonies are social and founded by a single female, diapause survival directly impacts springtime population sizes of foraging workers. Although *B. impatiens* is not currently in decline, diapausing insects undergo similar physiological shifts in preparation for diapause, these being developmental arrest, metabolic suppression and environmental stress resistance (Koštál *et al.*, 2017, MacRae, 2010). As such, physiological characteristics predicting diapause survival are likely to be broadly informative across bee species.

At the beginning of the life cycle, a single bumble bee queen founds a colony following a winter-diapause that can last up to 9 months, depending on both the species and geographical region (Alford, 1969b, Colla *et al.*, 2011). The colony develops progressively, producing workers until the end of the season when sexuals (queens and males) are produced (Amsalem *et al.*, 2015b). These young queens are thought to build up nutritional stores for several days post emergence, before exiting the colony to mate and seek a suitable hibernaculum for their winter diapause (Amsalem *et al.*, 2017, Amsalem *et al*., 2015b, Goulson, 2010, Woodard *et al*., 2019). In this study, we first examine the relationship between queen age at diapause onset and diapause survival length, hypothesizing that premature or delayed entrance into diapause results in reduced survival length. We then examine if and how pre-diapause body mass at two time points (upon eclosion and at the age of 7 days, when fat body reserves are assumed to be fully accumulated) correlates with diapause survival length, hypothesizing that heavier queens will have an increased length of survival. Unlike other studies (Beekman *et al*., 1998, Gosterit *et al.*, 2009), we did not test diapause survival using a pre-determined time point, but continuously monitored survival on a weekly basis until all queens died over a period of 350 days, a time period that is equivalent to the time queens spend in natural diapause. We then examined the role of two potential mechanisms mediating these effects: the acquisition of key nutrients and oxidative stress levels in pre-diapause queens of different ages, hypothesizing that young queens are lacking adequate nutritional stores to survive diapause, and that older queens will show higher levels of oxidative stress.

## Materials and methods

### Bumble bee rearing and collection

Queens (*n* = 419) were sampled from 16 bumble bee colonies obtained from Koppert Biological Systems (Howell, MI). Colonies were maintained in the laboratory in a walk-in incubator under total darkness, a temperature of 28–30°C and 60% relative humidity. All experiments were conducted under a red light. Colonies were supplied with unlimited food (60% sucrose solution and light spring bee pollen collected by honeybees, purchased from Swarmbustin’ Honey) during their entire developmental period. Fresh pollen was provided every 48 h. In all experiments, newly emerged queens (determined by a distinct silvery appearance) were separated from their parental colonies upon eclosion and individually tagged with a distinct colour and number (Opalith-Plättchen). Queens were left unmated to avoid introducing any confounding effects of mating. While mating is an important element of the diapause program (Baer *et al.*, 2005, Greeff *et al.*, 2008), previous studies showed that queens sequester fat body nutrients and enter and survive diapause regardless of mating status (Alford, 1969a, Amsalem *et al*., 2017, Costa *et al.*, 2020). Queens were then placed in plastic cages (11 cm diameter × 7 cm tall) where they were provided with *ad libitum* sucrose syrup and pollen and kept under the same conditions as above until the desired age of diapause onset.

### Experiment 1—the effect of queen age on diapause survival length

Individual newly emerged queens (*n* = 205) were sampled from four parental colonies and aged for 2–30 days in individual cages (3–16 queens for each age). Queens were sampled so that each parental colony was represented in the following four age categories: 2–6 days (*n* = 54), 7–12 days (*n* = 57), 13–17 days (*n* = 49) and 18 days or older (*n* = 45). Queens were then placed in 50-ml falcon tubes with mesh over the top to allow for ventilation, within cardboard boxes (28 × 22 ×5 cm) in cold storage conditions mimicking diapause (3–5°C, total darkness, and >70% relative humidity). High humidity was maintained by keeping a tray of ~3 cm of water in the bottom of the fridge at all times. Survival was checked weekly by exposing queens to room temperature (~25°C) and ambient light for 10 min and observing queens for movement. Queens that displayed movement were put back into cold storage, while queens displaying no movement were marked as dead (Amsalem *et al.*, 2015a, Fauser *et al.*, 2017, Treanore *et al.*, 2020). All queens were maintained for exactly 10 min outside the fridge regardless of when they showed movement.

### Experiment 2—the effect of body mass on diapause survival length

Newly emerged queens (*n* = 132) were sampled as above from four different parental colonies and aged in individual cages for 7 days (19–60 queens per colony). Queens were weighed using an electronic scale (Accuris Instruments, W3100A-120) immediately upon eclosion and again 7 days later prior to placing them in cold storage conditions as above. Survival was checked weekly as above.

### Experiment 3—the effect of queen age on nutrient acquisition and accumulation of hemolymph cryoprotectant

Newly emerged queens (*n* = 57) were sampled from six parental colonies and placed in individual cages as above (3–25 queens per colony). Hemolymph samples for protein and glycerol levels were taken from queens that were aged for 0, 2, 4, 6, 8, 10, 20, or 30 days (see below), after which queens were kept at 20°C for further body composition assays (lipid and glycogen, same-age groups as above). All queens were weighed, and their ovary activation was measured prior to analysis and included as a covariate in all body composition analyses (see below).

### Experiment 4—the effect of queen age on oxidative stress

Newly emerged queens (*n* = 26) were sampled from three parental colonies (8–10 queens per colony) and placed in individual cages as above. Queens were aged for 3, 12 and 24 days after which they were flash frozen on dry ice for 30 s for oxidative stress assays (see below).

### Analysis of protein and glycerol levels in the hemolymph

To extract hemolymph, live queens were anesthetized on ice until movement slowed. The cuticle was sterilized using 70% ethanol and a hypodermic needle (25GX5/8″) was inserted under the fourth abdominal segment (Amsalem *et al*., 2017). Hemolymph was extracted using a 10-μl glass capillary tube, and at least 6 μl was extracted from each individual queen and immediately placed on ice. For analysis of glycerol levels, 4 μl hemolymph was mixed with 6 μl PBS buffer and stored at −20°C until analysis. For analysis of protein levels, 2 μl hemolymph was mixed with 200 μl PBS buffer and stored at −20°C until analysis.

Glycerol levels were quantified by incubating the samples with 0.8 ml free glycerol reagent (Sigma-Aldrich, Inc., St Louis, MO, USA) at room temperature for 10 min. Following incubation, individual samples were read in triplicate on a NanoDrop One™ at 540 nm. Glycerol concentration was quantified using a standard curve calculated using a pure glycerol standard (Sigma-Aldrich, Inc., St Louis, MO, USA).

Protein levels were quantified by incubating 50 μl of the hemolymph/PBS mixture with 1.5 ml Bradford assay reagent for 10 min at room temperature. Following incubation, individual samples were read in triplicate on a NanoDrop One™ at 595 nm. Protein concentration was calculated from a standard curve using bovine serum albumin (BSA) standards.

### Bee dissection and analysis of ovarian activation

Following hemolymph extraction, queens were placed in −20°C until further analysis. Individual queens were thawed and dissected under a stereo-microscope. The ovaries were separated and scored by averaging the length of the three largest terminal oocytes. Queens have eight ovarioles (four in each side), and the terminal oocyte of at least one ovariole per ovary was measured with a scaled built-in ocular (Amsalem *et al*., 2015a). Following ovary dissections, the eviscerated abdominal fat body of individual queens was separated, weighted and placed in 1 ml 2% sodium sulphate for glycogen and lipid analysis.

### Analysis of glycogen and lipids in the fat body

Levels of glycogen and lipids were measured as in Amsalem *et al*. (2015a). The protocol was slightly modified to account for the high concentration and sensitivity of the spectrophotometer. In short, a quarter of the eviscerated fat body homogenate (250 μl) was further diluted by adding 4.75 ml 2% sodium sulphate. The homogenate was well mixed before 200 μl were sampled and combined with 2.8 ml chloroform/methanol mix (v:v 1:1). The samples were centrifuged to achieve separation between the precipitate (glycogen) and the rest of the solution. The two layers were separated, and glycogen was measured with the hot anthrone reaction (5 ml anthrone/sample). The remaining fraction was mixed with 2 ml of distilled H_2_O to achieve separation between the upper aqueous fraction (mostly free sugars) and the lower fraction (lipids). The two layers were separated, and lipids were quantified by a vanillin–phosphoric acid reaction (5 ml vanillin/sample). A standard curve for glycogen was created by using anhydrous glucose diluted in distilled H_2_O, and for lipids by using vegetable oil diluted in chloroform. Absorbance values (OD 630 for glycogen and OD 525 for lipids) for each sample were measured in triplicates with a NanoDrop One™ and converted to micrograms per queen based on the standard curve equation from each respective regression line. Dilution factors were included in calculating the final amount per sample. The amounts of glycogen and lipids are presented as concentration of μg/mg tissue mass, to normalize for differences in queen size.

### Oxidative stress

Oxidative stress levels were examined using a non-enzymatic component of the antioxidant system, glutathione (GSH) and the ratio of GSH to oxidized glutathione (GSSG). GSH/GSSG ratio and the amount of GSH and GSSG were examined in the thorax tissue of queens using a spectrophotometric GSH/GSSG assay kit (Eagle Biosciences). Thoraxes were separated from the rest of the body and weighed prior to analysis. To prevent oxidation, the thorax tissue was ground on ice in a glass tissue grinder with 1 ml 7.4100 mM Tris–HCL buffer, following a modified protocol by (Lalouette *et al.*, 2011). The homogenate was centrifuged at 4°C for 15 min until the supernatant was clear. Glutathione (GSH) and oxidized glutathione (GSSG) were measured in triplicate and measurements were taken every minute for 10 min at 412 nm using a BioTek Synergy LX PlateReader. GSH and GSSG contents were calculated from standard curves using the same kinetic measurement approach, and the ratio of GSH/GSSG was calculated using the following equation (GSH-2*GSSG)/GSSG) according to the manufacturer’s instructions.

### Statistical analyses

Statistical analyses were conducted using SPSS v.21 (IBM) software, and data visualizations were performed with the R package ‘*ggplot2*’ in R version 3.5.3 (R Development Core Team). Data were tested for normality prior to the statistical analysis and best-fitting models were selected using the lowest Akaike information criteria (AIC) value. We used a generalized linear mixed model (GLMM) with a normal distribution to examine the effect of queen mass on diapause survival length and report the estimate ± S.E.M of the fixed-effect parameter in the model. Parental colony was included as a random effect in the model. We used the curve estimation tool in SPSS to determine the best line of fit for examining the effect of queen age on diapause survival length. The effects of age on lipid, glycogen, protein and glycerol amounts were examined using a GLMM, using age as the fixed effect and oocyte size as a covariate; parental colony was included as a random effect but was not significant for any variables (*P* > 0.05). Oocyte size was analyzed using a GLMM with age as the fixed effect and parental colony as a random effect. Total glutathione (GSH) and oxidized glutathione (GSSG), as well as the GSH/GSSG ratio in the thorax tissue, were log-transformed and analyzed using one-way ANOVAs with queen age as the fixed effect. Post hoc pairwise contrast estimations using the least significant difference (LSD) method to control for multiple comparisons were performed between queen age groups in both the body composition and glutathione assays. Robust estimation was used to handle violations of assumptions in all models, and all GLMMs were performed on standardized values (*Z* scores). Data in figures are presented as boxplots featuring the minimum and maximum values, and outliers. Data in text are presented as means ± S.E.M. Statistical significance was accepted at *α* = 0.05.

## Results

### Experiment 1: the effect of queen age on diapause survival length

**Figure 1:**
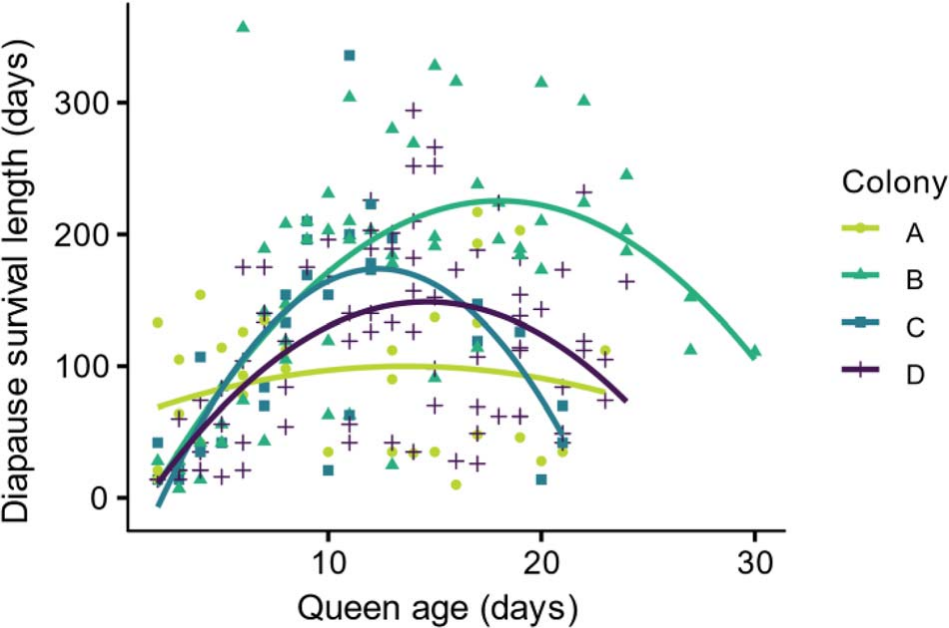
**The effect of queen age on diapause survival length in *B. impatiens* queens**. The number of days *Bombus impatiens* queens survived in diapause-like conditions (3–5°C and >70% relative humidity) as function of their age at the onset of diapause (*n* = 205 queens, **A**: *n* = 34, *R* = 0.17, *P* = 0.59, **B**: *n* = 58, *R* = 0.67, *P* < 0.001, **C**: *n* = 31, *R* = 0.74, *P* < 0.001, **D**: *n* = 82, *R* = 0.56, < 0.001)

Queens’ survival in cold storage was comparable to the length of natural diapause (6–7 months in *B. impatiens*) (Alford, 1969b, Colla *et al*., 2011). On average, queens survived 121 ± 5.67 days with substantial variability between queens, ranging between 7 and 350 days. When examining the effect of age on survival, a quadratic regression model (*R* = 0.49) proved to be a better fit than a simple linear regression (*R* = 0.35) based on lower AIC values (Fig. 1). Due to a parental colony effect on survival length (*P* < 0.001), each parental colony was analyzed separately. On average, queens from colony B survived the longest (161 ± 12 days) compared to queens from the other colonies (A; 88.1 ± 9.81 days, C; 110 ± 14.5 days, D; 111 ± 7.93 days). In three out of the four colonies we examined, there was a significant, quadratic effect of age on survival length (*r* = 0.67, *n* = 58, *P* < 0.001; *r* = 0.74, *n* = 31, *P* < 0.001; *r* = 0.56, *n* = 82, *P* < 0.001 for colonies B–D, respectively, Fig. 1). In colony A, there was not a significant relationship between age at diapause onset and survival length (*r* = 0.17, *n* = 34, *P* = 0.59). There was an optimum age range where survival length was maximized. Queens 5 days and younger survived for a shorter period of time compared to queens between the ages of 6 and 17 days. We also observed a slight decrease in survival length in queens older than 17 days.

### Experiment 2: the effect of body mass on diapause survival length in 7-day-old queens

**Figure 2:**
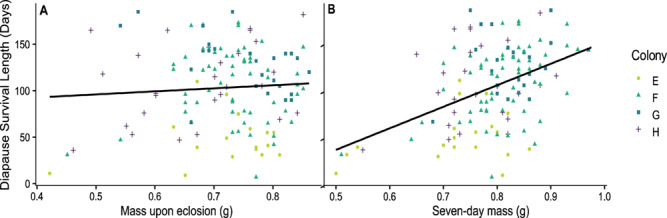
**The effect of body mass on diapause survival length in *B. impatiens* queens.** The number of days *Bombus impatiens* queens survived in diapause-like conditions (3–5°C and >70% relative humidity) as (**A**) a function of their mass upon eclosion (GLMM, *F*_1,130_ = 0.27, *P* = 0.61) and (**B**) their mass at the age of 7 days prior to diapause onset (GLMM, *F*_1,130_ = 21.036, *P* < 0.001). All queens were placed in diapause immediately after their body mass was measured (*n* = 131 queens)

On average, 7-day-old queens survived 102.7 ± 3.8 days in diapause and heavier queens survived longer compared to lighter queens (Fig. 2). Seven-day body mass of queens at diapause onset was a positive predictor of diapause survival length, while body mass at eclosion was not a significant predictor (for body mass of 7-day-old queens, GLMM, 0.33 ± 0.06, *F*_1,130_ = 21.036, *P* < 0.001; for body mass at eclosion: GLMM, 0.04 ± 0.078, *F*_1,130_ = 0.27, *P* = 0.61; Fig. 2). Parental colony did not have a significant effect on queen survival length (*P* > 0.25). During the first 7 days post-eclosion, queens increased their mass on average by 73.4 ± 7.4 mg (mean ± S.E.M) (*n* = 131), representing an average increase of 10% in their body mass. Over the course of diapause, queens lost on average 219 ± 7.7 mg (mean ± S.E.M) during the average time period listed above (*n* = 131), representing an average loss of 28% of their body mass. Average mass changes were calculated using the average mass of queens upon emergence vs. 7 days of age and upon diapause termination vs. diapause onset.

### Experiment 3: the effect of queen age on nutrient acquisition and accumulation of hemolymph cryoprotectant

**Figure 3:**
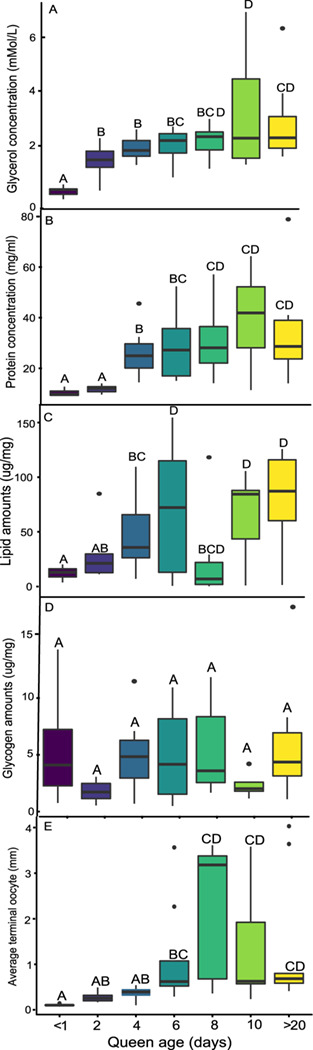
**The effect of age on macronutrient accumulation and ovary activation in *B. impatiens* queens.** Concentration of (**A**) glycerol (*n* = 55) and (**B**) protein (*n* = 57) in the hemolymph, and amount of (**C**) lipids (*n* = 57) and (**D**) glycogen (*n* = 57) in the fat body. (**E**) Average terminal oocyte size (mm) of *Bombus impatiens* queens at seven timepoints between < 24 h post-eclosion and 20–30 days of age. Different letters indicate statistical significance at *α* = 0.05. Post hoc pairwise contrast estimations were performed using least significant difference (LSD)

Glycerol and protein concentration in the hemolymph increased with age in queens following pupal eclosion (Fig. 3). Protein concentration was significantly affected by age (GLMM, *F*_6,49_ = 13.04, *P* < 0.001) as well as the average oocyte size (GLMM, *F*_1,49_ = 22.37, *P* < 0.001). Post hoc analyses show that queens younger than 2 days old had a significantly lower concentration of hemolymph proteins (<11.8 ± 0.57 mg/ml) compared to the other age groups (Fig. 3). Ten-day-old queens had the highest concentration of protein (39.7 ± 6.95 mg/ml), which was not significantly different than the concentration in queens 8 days of age or older. The concentration of glycerol in the hemolymph was significantly affected by queen age (GLMM, *F*_6,47_ = 6.04, *P* ≤  0.001), but not by the average oocyte size (GLMM, *F*_1,47_ = 2.88, *P* = 0.10). The post hoc analyses showed that queens younger than 1 day of age had significantly lower glycerol concentration (0.34 ± 0.07 mM/L) compared to the other age groups. However, within 2 days, the glycerol concentration was almost five times higher (1.49 ± 0.25 mM/L). Ten-day-old queens had the highest concentration of glycerol; however, this was not significantly different than the glycerol concentration in 8-day-old queens (Fig. 3).

The amount of fat body lipids in queens was significantly affected by age (*F*_6,49_ = 6.20, *P* < 0.001) and by the oocyte size (*F*_1,49_ = 17.97, *P* < 0.001). Fat body lipid amounts increased with queen age, with 12.3 ± 2.03 μg/mg fat body lipids in queens younger than 1 day of age compared to 75.4 ± 16 μg/mg in queens older than 20 days of age. We further observed an unexpected decrease in fat body lipids in 8-day-old queens, which although not differing statistically from the age groups above 6 days may have been caused by an increase in ovarian activation in that age group (Fig. 3). Age did not have a significant effect on fat body glycogen content (*F*_6,49_ = 1.89, *P* = 0.10), but oocyte size did (*P* = 0.003).

The average oocyte size was significantly affected by age (GLMM, *F*_6,46=_5.06, *P* < 0.001) but not by parental colony (*P* = 0.26; Fig. 3). Queens younger than 4 days of age had the smallest average oocyte size (<0.37 ± 0.43 mm), and 8-day-old queens had the largest oocytes (2.18 ± 0.58 mm). Post hoc comparisons between age groups show that queens younger than 1 day of age had significantly smaller oocyte size as compared to all queens older than 4 days of age (*P* < 0.001). There were no significant differences in oocyte size among queens older than 6 days of age.

### Experiment 4: the effect of queen age on oxidative stress

**Figure 4:**
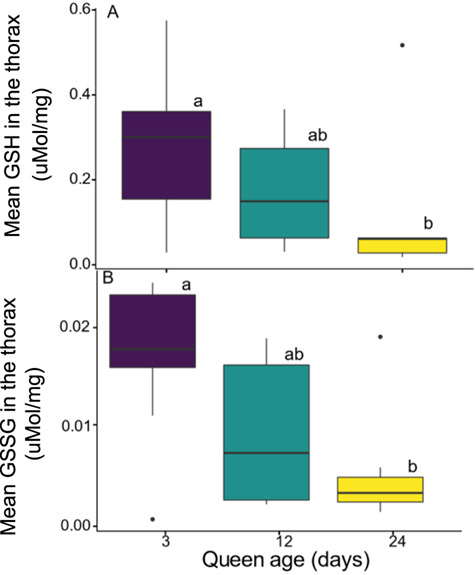
**The effect of age on oxidative stress in *B. impatiens* queens.** Average levels of (**A**) GSH and (**B**) GSSG (μM/mg fresh mass) in the thorax of *Bombus impatiens* queens at 3, 12 and 24 days of age (*n* = 26). Different letters indicate statistical significance at *α* = 0.05. Post hoc pairwise tests were performed using least significant difference (LSD)

Both GSH and GSSH levels in the thorax tissue decreased with queen age, and both were significantly affected by age (one-way ANOVA, GSH, *F*_2,23_ = 3.80, *P* = 0.04; GSSG, *F*_2,23_ = 3.40, *P* = 0.05, Fig. 4). Parental colony did not have a significant effect on GSH/GSSG content, or GSH/GSSG ratio (*P* > 0.05). Post hoc analyses show that 3-day-old queens had significantly higher amounts of GSH in their thorax tissue as compared to 24-day-old queens (*P* = 0.01). The GSH levels in the 12-day-old group were intermediate and did not differ significantly from the other age groups (*P* > 0.10). Post hoc comparisons of GSSG amounts in the thorax followed a similar pattern, with significantly higher amounts of GSSG in 3-day-old queens compared to 24-day-old queens (*P* = 0.02) and intermediate insignificant levels in the 12-day groups compared with the other age groups (*P* > 0.09). Comparisons of the GSH/GSSG ratio in the thorax tissue of queens of different ages showed no significant differences between the age groups (*F*_2,23_ = 0.03, *P* = 0.97).

## Discussion

Bee declines have been linked to a number of extrinsic ecological factors including limited dietary diversity, specific habitat requirements (e.g. alpine forests), and late-season female diapause emergence, which may limit nest site availability (Bartomeus *et al.*, 2013, Colla *et al.*, 2012, McFrederick *et al.*, 2006, Williams *et al.*, 2009). However, the intrinsic traits in bees interacting with these ecological factors are less well-studied. Here, we examined physiological factors affecting diapause survival length in *B. impatiens* queens. We hypothesized that mass and age would both affect survival, with higher mass and mid-age ranges of queens surviving the longest. We propose that this is primarily driven by timing of nutrient acquisition post-eclosion and the effects of stressors associated with aging in older queens.

Queens within the middle range of ages, ~6–17 days of age, survived the longest, which supports our hypothesis about an optimum timeframe for entering diapause. The survival length of young queens, <6 days of age, was the lowest, likely attributed to insufficient nutritional stores. Queens 18 days or older showed a slight decrease in survival length, supporting our hypothesis about decreased survival due to stressors related to aging. Together with findings from other studies, these data show that shifts in queens’ physiological quality, as well as the interaction between nutritional status and age (Costa *et al*., 2020), may influence diapause survival. While the queens in our study were unmated (Baer *et al.*, 2005, Colgan *et al.*, 2019), previous research in *B. terrestris* queens found that, on average, unmated queens survived only 5 days longer as compared to mated queens in cold storage (Greeff *et al*., 2008). Therefore, the lack of mating is unlikely to significantly affect our data on queen survival. Additionally, we found mass to be a positive predictor of diapause survival, with heavier queens surviving longer periods of time. Previous studies in bumble bees, which examined the effect of pre-diapause mass on diapause survival, found no correlation between body mass and the length of survival excluding a threshold under which queens are unlikely to survive diapause (Beekman *et al*., 1998, Vesterlund *et al.*, 2014), used limited categorical data or did not control for queens age (Bogo *et al*., 2017, Gosterit *et al*., 2009, Holm, 1972, Owen, 1988). These studies looked at survival at a predetermined timepoint, unlike our study where survival was monitored continuously on a weekly basis until all queens died for a period equivalent to natural diapause. Longer survival due to higher mass is likely linked to increased nutrient reserves. The nutrients that increased the most post-eclosion were lipids in the fat body, and glycerol and proteins and in the hemolymph. Lastly, our examination of oxidative stress showed a reduction in the reduced and oxidized forms of glutathione. Overall, our results suggest that survival length of diapause is regulated not only by environmental (extrinsic) factors, such as access to nutritional and habitat resources, but also by underlying physiological (intrinsic) factors, and possibly also by the interplay between the two.

Findings from this study emphasize the importance of access to floral resources, particularly for queens post-eclosion. Bumble bee declines have been linked to nutritional stressors (e.g. reduced floral resource availability) (Goulson *et al.*, 2008) and reliance on a narrower suite of host plants (Bartomeus *et al*., 2013, Wood *et al.*, 2019). The correlation between body mass and survival in 7-day-old queens highlights the importance of mass acquired post-eclosion for a longer diapause survival. For example, species relying on narrow dietary range (herbaceous plants) and experiencing partial range declines, such as *B. pensylvanicus* (Wood *et al*., 2019), may face challenges in gaining sufficient mass pre-diapause. This problem is not limited to species with narrow diets, as even bumble bee species with a wider dietary breadth may suffer from limited resource availability post-eclosion due to habitat destruction and phenological mismatch caused by climate change (Goulson *et al*., 2008, Pyke *et al.*, 2016). Furthermore, mass loss during diapause can be exacerbated by exposure to neonicotinoids pre-diapause (Fauser *et al*., 2017), suggesting that limited floral resources and pesticide exposure can reduce diapause survival.

Access to floral resources is critical for the accumulation of key nutrients post-eclosion (Alford, 1969b, Woodard *et al*., 2019), but so is the quality of these resources. Floral resources differ significantly in the nutritional quality of their pollen (Vaudo *et al.*, 2020) and nectar (Baker *et al.*, 1983), and both are necessary for nutrient accumulation pre-diapause (Woodard *et al*., 2019). Additionally, nutritional status in *B. impatiens* queens has been suggested to affect diapause entrance (Costa *et al*., 2020). We examined nutrient accumulation in post-eclosion queens with access to *ad libitum* pollen and nectar by looking at lipids and glycogen in the fat body, and glycerol and protein in the hemolymph.

Lipids, a critical fuel source during insect diapause (Hahn *et al*., 2011), increased in their amount in the fat body of queens post-eclosion and were likely derived from pollen, the main source of dietary lipids (Roulston *et al*., 2000). Lipids are primarily stored as triglycerides and are important for diapausing individuals due to their high energy yield and the high amount of metabolic water they provide (Hahn *et al*., 2011). The amount of lipids in the fat body primarily increased in newly emerged queens (<1 day old) until around 6 days old, at which point lipid content in the fat body plateaued. Our results support previous studies suggesting that lipid acquisition occurs soon after eclosion (Alford, 1969a, Woodard *et al*., 2019). While lipid amounts generally increased with age, we observed a decrease in lipid amounts in 8-day-old queens, which corresponded with an increase in ovarian activation in this age group. In the wild, newly emerged queens will not activate their ovaries until after diapause (Amsalem *et al*., 2015b). However, in captivity, queens will often do so following separation from the parental colonies and in the presence of unlimited food supply (Amsalem *et al*., 2015b). Fat body nutrient depletion to sustain reproductive processes is a frequently observed trade-off in insects. *Osmia rufa*, for example, have exhibited similar transitions in nutrients from the fat body to the ovaries (Wasielewski *et al.*, 2011), and *B. impatiens* queens demonstrated similar reallocations of nutrients following transition from a pre-diapause state to reproduction (Amsalem *et al*., 2017).

Queens emerged with a substantial amount of glycogen already stored in the fat body upon eclosion, suggesting glycogen is likely to accumulate during the developmental stages, in line with findings from other holometabolous insects (Tolmasky *et al.*, 2001). Glycogen amounts varied over the time period we observed, suggesting that high amounts of glycogen are not critical for diapause survival, nor are they a limiting factor during early adulthood. These fluctuations could also be related to the ovarian activation we observed in some age groups, similar to what was previously found in *B. terrestris* (Roseler *et al*., 1986). Through glycolysis, glycogen can be broken down into glycerol precursors (triose phosphates) during cooler periods of diapause (Joanisse *et al*., 1995), suggesting glycogen may be used as a secondary reserve during a longer diapause, as often observed in insects living in seasonal environments (Hahn *et al*., 2011). We further found that hemolymph glycerol concentration increased pre-diapause, with higher concentration in old compared to younger queens, suggesting that queens begin producing glycerol soon after eclosion as a physiological adaptation to survive cold temperatures associated with diapause. These results are in line with previous work in *B. terrestris* indicating they are a freeze-avoidant species (Owen *et al.*, 2013), meaning they employ physiological and behavioural adaptations to endure freezing temperatures. Additional research examining glycerol (and other cryoprotectants such as proline or trehalose) concentration over the course of diapause or with fluctuating temperatures could elucidate if these levels remain steady during diapause or if they increase with additional cold exposure.

Finally, protein concentration in the hemolymph significantly increased with age in queens, yet the exact function, source and identity of these proteins are unknown. The family of hexamerin storage proteins is a likely source. Hexamerins are often accumulated in pre-diapause insects to serve as a source of amino acids and energy during diapause (Hahn *et al*., 2007). Other proteins that often increase prior to diapause are a large family of heat shock proteins that are produced in response to exposure to cold temperatures (Denlinger, 2002). Both types of proteins are critical for dealing with stressors associated with diapause, such as cold stress and starvation. However, a more detailed analysis of the proteins present in the hemolymph pre-diapause [see (Colgan *et al*., 2019)] could be informative to understanding which stressors queens are equipped to mitigate during diapause (e.g. temperature stress or pathogens) and which stressors are more likely to result in queen mortality.

Our examination of age, diapause survival length and oxidative stress highlighted key physiological shifts that occur in pre-diapause queens. Young queens (<6 days) lacked sufficient nutrient reserves and thus experienced high rates of mortality. Our results examining nutrient acquisition confirmed this, showing the ages when nutrient stores plateaued. In older queens, we also observed decreased survival that was not explained by insufficient nutrient stores. Thus, we examined the hypothesis that older queens had higher levels of oxidative stress. Our results were mixed, as we found an overall decrease in glutathione levels with age, but no decrease in the GSH:GSSG ratio.

While reduction in the reduced or oxidized form of glutathione may indicate oxidative stress, stronger evidence would be a further decrease in the GSH:GSSG ratio. However, the absence of this could be explained by the experimental design, particularly in having the queens reared in captivity with limited access to flying. Flight is one of the most energetically costly activities in flying insects, producing reactive oxygen species (ROS) that cause oxidative damage in thoracic flight muscles (Fisher-Wellman *et al.*, 2009, Levin *et al.*, 2017). Furthermore, the lack of environmental stress in our experimental design (i.e. constant temperatures and high relative humidity and unlimited food) could also explain the low amounts of GSH that were found. Study in honey bees (*Apis mellifera*) found that infection with pathogens (*Nosema ceranae*) resulted in increased activity of GST (glutathione transferase) and higher amounts of GSH (Łopieńska-Biernat *et al.*, 2017). Additionally, honey bee queens of various ages exhibited decreased expression levels of *gst1* (a gene encoding to an oxidative enzyme; (Corona *et al*., 2005)). Examining oxidative stress at multiple regulatory levels (gene, protein) and using external stressors (e.g. allowing flight in larger arenas) before and during diapause may further elucidate their importance for diapause survival.

Our study emphasizes how underlying physiological factors can predict queen survival of diapause. It also demonstrates how ecological factors associated with bee declines are connected to intrinsic traits. Species with shorter periods of adult activity or narrow dietary requirements prior to diapause may be the most vulnerable to external stressors, with queens at risk of entering diapause in suboptimum physiological condition. For example, in declining species like *B. affinis*, queens emerge later in the season (August in eastern North America) and experience a shorter activity period of adulthood prior to diapause (Williams *et al*., 2009, Wood *et al*., 2019), a characteristic that may lead to decline through its effect on diapause mortality. Late-season resource dearth has been suggested as a factor contributing to species decline in a number of recent studies (Balfour *et al.*, 2018, Timberlake *et al.*, 2019, Wood *et al.*, 2018), with bumble bee queens being most vulnerable to these resource shortages.

Nonetheless, numerous conservation strategies can be used to reduce mortality during diapause. Agricultural landscapes are frequently characterized by limited temporal or spatial floral resource availability, but the implementation of flowering strips (Rundlöf *et al.*, 2014), hedgerows (Kremen *et al.*, 2019) or flowering cover crops (Mallinger *et al.*, 2019) can offer late-season forage. These should be optimized to complement periods of resource dearth and provide forage for the entirety of the pre-diapause period of different species. Maintaining habitat connectedness and abundant floral richness can reduce the likelihood of oxidative damage or predation resulting from increased pre-diapause foraging. Additionally, protection of preferred diapause habitat (e.g. soil type, moisture content) is necessary to ensure that queens can find suitable nesting sites and enter diapause in optimal physiological conditions. Yet, this relies on continued characterization of queen diapause habitats (Williams *et al.*, 2019), pre-diapause diet and life history of bumble bee species, especially those in decline. Through retention of late-season floral resources and integration of research on intrinsic physiological traits and extrinsic ecological factors, survival of the diapause life stage can be improved in annual bee species.

## Funding

This work was supported by the United States-Israel Binational Agricultural Research and Development Fund [US-5182-19 awarded to EA], by the Apes Valentes Research fund [awarded to EDT] and by the USDA National Institute of Food and Agriculture [USDA NIFA AFRI Predoctoral fellowship program #12904957 awarded to EDT].
